# Supplementary material for: Are carbon emissions trading and green financial instruments synergistic? -Comprehensive quantitative research based on content analysis
Source: PLoS One. 2024 Mar 7;19(3):e0298601. doi: 10.1371/journal.pone.0298601 (PMC10919601; doi:10.1371/journal.pone.0298601)
Supplement: S1 Data — (ZIP) [file pone.0298601.s001.zip › Data/地级市Z_F工作报告.zip!/║┌┴·╜¡/╞▀╠¿║╙╩╨/└┤╫╘╣1⁄2╓┌║┼í╛┬φ┐╦╩2╛▌═°í┐_2013.docx]

各位代表：

　　我代表市人民政府向大会作工作报告，请予审议。并请市政协委员和各位列席人员提出意见。

　　一、2012年工作回顾　　过去的一年，在国内外宏观经济环境影响下，煤焦等原材料市场萎缩、价格下降，我市经济社会发展遇到前所未有的困难。在省委、省政府和市委的领导下，在市人大和市政协的监督支持下，我们全力上项目、稳增长、抓改革、惠民生，虽然经济增长低于年初预期目标，但发展呈现出稳中有进的良好态势。

　　经济实现平稳增长。经济发展速度高于上年。预计全市生产总值实现303 亿元，增长7.5％，增幅比上年提高2.2 个百分点。规模以上工业增加值139 亿元，增长8％，增幅比上年提高0.8 个百分点。经济发展质量有所提高。公共财政预算收入21.8 亿元，增长6.9％。城乡居民收入高于经济增速，城镇居民人均可支配收入17900元，农民人均纯收入8980元，均增长9％。经济发展基本面向好。固定资产投资快速增长，完成投资163 亿元，增长35.5％。内外贸易比较活跃，社会消费品零售总额73 亿元，增长15％；外贸进出口总额1 亿美元，增长8％。

　　产业发展基础增强。产业项目建设迈出新步伐。严格落实产业项目包保责任制，促进项目洽谈签约、审批落地、开工建设。完成工业固定资产投资83 亿元，增长35％，占全社会固定资产投资50.9％。新建续建千万元以上产业项目64 个，其中，亿元以上项目26 个，竣工7 个。煤化工产业链条延伸，宝泰隆和龙盛达两个30 万吨煤焦油深加工项目、隆鹏10 万吨甲醇项目试产，金泰6 万吨环己酮和5 万吨己二酸、宝泰隆5 万吨针状焦项目建设顺利。战略性新兴产业开始起步，国家“973”重点攻关项目成程非晶合金等高新技术产业项目开工建设。重点园区建设取得新进展。对上争取政策资金支持，再生资源回收利用产业园区成为全省唯一的国家城市矿产示范基地，煤化工产业园区晋升为省重点产业园区，经济开发区基础设施建设投入1.1 亿元，引进项目10 个，完成投资2.4 亿元。招商引资实现新突破。33 个专业招商组常年驻外招商，引进5000 万元以上产业项目22 个，完成投资3 亿元。启动产业项目储备之冬会战，重奖重用引资有功人员，干部群众招商积极性明显增强。

　　“三农”工作扎实推进。种植业喜获丰收，克服旱、涝、风等灾害影响，粮食产量达到17.5亿斤，实现九连增。畜牧业规模化发展，新增标准化畜牧养殖场48 个。产业化程度稳步提升，大兴牧业肉鸭屠宰加工、利健甜菊糖等省级龙头加工项目建成投产。小城镇建设试点稳步推进，新建住宅小区62 万平方米。新农村建设取得实效，投入资金1.3 亿元，实施基础设施、社会事业等项目102 个，解决40 个村屯、3.44 万人饮水安全问题。

　　民生事业加快发展。安居工程建设实现历史性跨越。新开工和续建保障性住房382 万平方米，竣工174 万平方米。其中，煤矿棚改工程交付房源35086 套，已安置10086 套；完成300套公租房、333 套经济适用房建设；新建4600套廉租房、5371 套城市棚改房。改造农村泥草房5000 户、危房513 户。社会保障能力稳步提高。城镇职工医疗保险金最高支付限额提高3万元、达到9 万元，医保购药日限额提高30 元、达到100 元，大病统筹医疗保险金支付上限提高8 万元、达到18 万元，城镇居民医疗保险金最高支付限额提高2 万元、达到5 万元，新农合补助标准提高40 元、达到290 元。失业保险金支付标准提高160 元、达到650 元。城市低保标准提高52 元、达到310 元。就业形势良好。新增就业1.3 万人，城镇登记失业率低于省控指标。教育、卫生事业长足发展。启动新一中建设，完成2 所学校教学楼、2 所学校宿舍楼主体工程，建成7 所公办幼儿园，办学条件进一步改善。市中医院、市精神病医院主体工程完工，市人民医院外科综合楼加快建设，新建改造6 个社区卫生服务中心、55 个农村卫生院所，医疗服务能力进一步提升。便民工程深入实施。开通民生“110”服务热线，受理解决民生问题984个。公路客运总站交付使用，更新环保公交车辆90 台、长短途客运车辆46 台。

　　城市建设管理加强。国家园林城市建设扎实推进。新增、改造绿地196 公顷，推进石油大道景观、学府街绿化带提档升级，完成主要街路绿化，改造提升59 个公园广场、住宅小区、厂区矿区绿化景观，全面完成国家园林城市整改达标任务，顺利通过国家专家组评审。“十项整治”活动成效斐然。多渠道筹措资金11.9 亿元，实施整治项目358 个。开工建设大同街东延工程，完成石油大道东延、东进街等6 条道路改造、12 项给排水管网改造项目，城市功能进一步完善。整饰楼体立面132 栋，完成4 条街路亮化工程，集中整治4 条街路牌匾，综合整治179 个住宅小区、厂区矿区环境，实施3 个矿山环境恢复治理项目，城市面貌焕然一新。城市管理长效机制初步形成。调整优化城市管理职能，完善细化“六化一考”城市管理制度，市场化运作率、环卫保洁率、绿化成活率稳步提高，道路交通秩序、客运市场秩序持续好转。节能减排成效明显。完成倭肯河流域及桃山水库污染综合整治项目12 项，实施国控污染源治理项目7 项和重点减排工程14 项，集中治理锅炉烟尘、汽车尾气、涉煤企业粉尘，减排烟尘粉尘4.3 万吨，万元GDP 能耗下降3.6％。城市供水危机有效缓解。争取国家和省资金支持，新打应急水源井101 眼，实行限时、限量、限制供水，平稳度过供水紧张时期。

　　社会形势总体稳定。信访工作成效明显。深入开展领导干部接访和机关干部下访、重点信访积案清理等活动，实现“信访总量、进省非正常访、突出信访问题”三下降。社会治安持续好转。实施“天眼”工程，开展“警灯闪烁”等活动，人民群众安全感增强。安全生产形势稳定。地方煤矿事故起数、死亡人数分别下降26.3％、26.7％。

　　科学执政能力提高。发展环境进一步优化。扎实开展“庸懒散慢”专项治理行动，精简行政审批项目6 项，取消涉企收费33 项，缓征焦化企业“一费一金”1580 万元，“双优支持工程”为企业融资2 亿元。依法行政水平进一步提高。深入开展“六五”普法活动，严格落实市人大及其常委会的决议决定，主动接受人大依法监督和政协民主监督，人大代表议案、意见、建议和政协委员提案办复率达到100％。各项重点改革进一步深化。强镇扩权等改革有序推进，安全生产监管体制等改革基本完成，城市管理体制等改革取得成效。

　　统计、审计、科技、外侨、食品药品、计生、文化、体育、老龄、气象、地震、人防、地方志、档案、民兵、预备役等工作取得新进展，民族宗教、妇女儿童、残疾人、红十字等事业取得新进步。

　　各位代表，过去的一年，我市在困境中实现的经济社会平稳发展来之不易。这是省委、省政府和市委正确领导的结果，是市人大、市政协和社会各界鼎力支持的结果，是全市上下拼搏实干的结果。在此，我代表市政府向各位代表和全市人民，向所有关心支持七台河发展的各界人士，表示崇高敬意和衷心感谢！

　　我们也清醒地认识到存在的问题。地区生产总值、公共财政预算收入、城乡居民收入分别低于预期4.5、9.1、3 个百分点，完成“十二五”规划目标难度加大；经济总量小，发展速度慢，产业结构单一，发展后劲不足；煤炭资源减量加快，城市经济转型较慢，土地等生产要素相对紧缺，节能减排、环境保护任务繁重，实现可持续发展压力增大；城市基础设施建设滞后，供水等公共服务难以满足加快发展需要；“12·1”矿难的发生，深刻反映出安全生产领域还有一些深层次的问题亟待解决；政府自身建设还不能适应转型发展的需要，工作绩效有待进一步提高，创新能力有待进一步增强，一些重点领域腐败问题仍不同程度存在。

　　二、2013年政府工作任务

　　2013 年是全面贯彻落实党的十八大精神的开局之年，是实施“十二五”规划承上启下的关键之年，是全面建成小康社会奠定坚实基础的重要一年。全球经济发展仍存在诸多不确定性因素，国内外市场需求不旺，煤焦等资源品供求、价格难有大的改变，我市经济增长、财政增收、就业保障、下游产业发展压力依然巨大，实现转型发展的难度增加。保障性住房、公路建设等政府还债压力加大，支撑产业项目建设、民生事业发展的财力更加紧张。但是我市仍处于大有作为的重要战略机遇期。国家把扩大内需作为长期战略方针，继续实施积极的财政政策、稳健的货币政策，增加财政赤字和国债规模，强化稳增长、调结构、促改革、惠民生政策措施；省委、省政府在产业项目三年攻坚决战之年，对重大产业项目和重点园区扶持力度加大，对民生事业发展投入加大，对产能过剩产业调整补贴加大；近两年我市所做的打基础、攒后劲工作成效逐步显现。这些发展机遇和良好条件，有利于我市经济提质增速。我们既要增强忧患意识，努力把各种不利影响降到最低；更要坚定必胜信心，努力把各种积极因素用到最好，促进经济社会持续健康发展。

　　2013 年政府工作的总体要求是：全面贯彻落实党的十八大、中央和省委经济工作会议、市委八届三次全会精神，以邓小平理论、“三个代表”重要思想、科学发展观为指导，围绕四个发展，更加注重产业项目建设调结构，更加注重城乡统筹发展促“三农”，更加注重城市建设管理提形象，更加注重保障改善民生增福祉，更加注重社会管理创新保稳定，全力促进城市经济转型，努力建设幸福之城。主要预期目标是：全市生产总值增长8％以上，规模以上工业增加值增长9％以上，公共财政预算收入增长5％以上，全社会固定资产投资增长30％以上，社会消费品零售总额增长15％以上，外贸进出口总额增长10％以上，城镇居民人均可支配收入和农民人均纯收入均增长10％以上。

　　（一）全力抓好产业项目建设，促进城市经济转型。围绕“依托煤、延伸煤、不唯煤、超越煤”发展思路，突出引进重大产业项目，力争亿元以上项目达到15 个，千万元以上项目达到64个，完成投资61 亿元。一是加快发展煤化工产业。谋划发展以焦炭、煤气、焦油、粗苯为原料的合成化工、精细化工、材料化工项目。加快金泰化工等在建项目建设，促进龙盛达等项目投产达效，推进甲醇制烯烃、芳烃等项目生成，积极开发乙烯、丙烯等下游产品，构建现代煤化工产业体系。二是大力发展战略性新兴产业。积极创造现代产业成长发展环境，加强与高等院校、科研院所合作，大力引进科研成果转化项目，促进德彩光电等在建项目投产达效，开工建设石墨深加工等项目，围绕非晶合金材料引进开发下游产业，构建现代产业体系。三是积极发展非煤优势产业。发挥畜牧业发展环境好、成本低、品质高的比较优势，大力引进畜产品深加工项目，带动畜牧养殖规模化发展，形成完整的畜牧业产业链。积极引进矿山机械制造、制药、木制品加工等企业，开工建设节能门窗和玻璃幕墙等项目，壮大优势产业规模。四是促进服务业加快发展。加快建设城市综合体项目，大力引进高档酒店、购物商场，积极发展大型连锁超市，构建和完善消费市场环境，促进消费增长。研究建设现代物流产业综合体，加快推进新兴现代物流园区等项目，打造黑龙江省东部物流产业基地。大力发展电子商务、旅游等现代服务业，着力引进咨询、评估、工程设计等生产性服务业。依托居民集中区建设，完善商业服务体系，适应城镇化发展需要。

　　（二）突出重点产业园区建设，增强项目承载能力。本着突出重点、促进产业集聚发展、集约利用土地的原则，加快建设重点产业园区。一是进一步明确园区发展定位。立足打造国家级产业园区，推进经济开发区基础设施、项目层次、投资强度提档升级。立足打造省重点产业园区，利用煤矿棚改腾空土地，规划煤化工产业园区，配套建设基础设施，积极发展现代煤化工项目，走出一条老工业区发展的新路子。立足打造国家城市矿产示范基地标杆，高标准规划建设再生资源回收利用产业园区基础设施，着力引进再生资源深加工大型企业，促进废弃电子电器储藏拆解利用项目落地，加快建设交易市场，形成较为完善的回收利用体系，提高园区项目集聚能力。以畜产品和果蔬加工为主导，谋划建设绿色食品加工产业园，形成黑龙江省东部重要的绿色食品加工基地。加快勃利县产业园区建设，促进县域经济健康发展。二是进一步创新园区管理体制机制。制定产业园区全市共建共用、产业项目全市统筹摆放、财政税收市县区合理分享的办法，强化产业园区管理团队建设，采取BOT、BT 等模式，加快园区基础设施建设，促进产业园区专业化、规模化发展。研究设立产业园区经济建设公司，搭建园区投融资平台，增强产业园区自我建设、自我发展、自我完善能力。三是进一步强化园区招商措施。严格落实领导带头招商责任制，发挥示范带动作用；调整充实专业招商力量，发挥招商主力军作用；落实部门招商任务，激发干部职工招商动力；重奖引资有功人员，激励全员参与招商；突出委托“外援”招商，聘请外地能人为我市引进项目，力争园区项目建设实现较快发展。

　　（三）加快发展特色农业，提高城乡一体化水平。坚持走绿色发展、特色发展、产业发展、规模发展之路，全力搞好小城镇建设，进一步缩小城乡差距。一是发展优势特色农业经济。推广玉米大垄双行栽培技术，连片耕地种植面积达到1.4 万亩以上，引导种植绿色特色、高产高效经济作物，粮食产量稳定在15 亿斤以上。大力发展城市农业，促进棚室果蔬产业发展。坚持组织、培训、就业、维权“四位一体”的劳务输出模式，实现劳务收入8 亿元以上。重点培育扶持养殖大户，新增标准化养殖小区10 个，大力提高畜牧业占农业经济的比重。加快引进龙头深加工企业，逐步实现农副产品初加工向规模化深加工转变。二是积极构建新型农业经营体系。引导建立以资金、技术、劳务和农产品营销等为纽带的专业合作组织和股份合作组织，统一生产销售，打造畅销品牌，增加种植养殖效益。支持农民发展农机合作组织，新增大型农机合作社5 个。发展多种形式的土地适度规模经营，加快土地向合作经济组织、种植大户、种田能手集中。三是加快城乡一体化进程。高标准完成小城镇建设规划，深入推进2 个省级百镇建设试点和5 个市级小城镇建设试点，完成英伦小镇一期工程，加快金沙新区基础设施建设，改造城中村5 个，整体搬迁行政村3 个，新建农民公寓27 栋，强化环境综合整治，全力促进新农村建设。

　　（四）切实加强城市建设管理，提升综合竞争能力。按照建设秀美七台河的发展定位，完善城市总体规划和专项规划，巩固国家园林城市创建和“十项整治”成果，增强城市发展承载能力。一是加强基础设施建设。实施大同街东延、景丰路南延、步行街和乌金桥、东进桥等新建改造工程，推进“三供”管网、雨水管网、应急水源等工程，启动连接七桦路和石油大道的城市西环路、依宝公路万宝公园至向阳段改扩建工程前期工作。综合评估、科学论证桃山水库二期和跨区域调水工程，尽快完善方案、推进实施，突破城市发展瓶颈。二是全面推进城区绿化建设。实施全城绿化工程，推进巷路、小区、厂区矿区、办公楼区等绿化，提升主要街路、广场公园、节点景观、城市出入口绿化品质。严格落实“绿色图章”制，新建工程同步完成绿化任务，实施北岸新城配套绿化工程。加强桃山水库绿化建设，增强涵养水源能力。三是提升城市管理水平。完善“六化一考”城市管理机制，细化城市管理标准，加快市政设施维护、城市绿化养护、环卫保洁等市场化进程。切实强化供热、供气等市场管理，依法惩处损害用户权益行为。完善数字化基础设施，建设智慧城市。建立城管、公安联合执法和部门联动服务机制，提高“12319”指挥平台管理服务效能。四是强化环境污染治理。实施重点工业污染减排工程10 项，限期治理超标排放工业锅炉，严格控制汽车尾气超标排放，力促涉煤企业上齐防尘网，焦化企业脱硫达标。实施垃圾处理场改扩建工程，加快第二污水处理厂建设，强化污水处理厂运行监管，促进医疗废物处置中心正常运营。推进倭肯河流域治理和矿山环境恢复治理。

　　（五）深化体制机制改革，增强发展内生动力。抓住影响和制约发展的关键环节，着力构建充满活力、富有效率、更加开放的体制机制。一是推进重点领域改革。实施厂办大集体改革，妥善处理企业债权债务，职工全部纳入社会保障体系。推进强镇扩权改革，赋予乡镇相应的行政、社会管理权，扩大乡镇财税权和人事调配管理权。推行街道社区体制改革，合理调整社区布局，减少管理层级，提升服务水平。完善煤炭税收征管体制，改革传统计量方式，创新票据核算系统，强化稽查监管，实现煤炭税收征管规范化。调整市区分税制政策，合理划分事权财权，调动各区发展积极性。深化司法、事业单位、医药卫生等专项改革。二是促进中小微企业健康成长。研究制定扶持政策，降低中小微企业准入门槛和收费标准，实行“试营业”、“一元注册”制度，搭建动产抵押、股权质押、银行信贷融资平台，着力创造全民创业、中小微企业竞相发展的良好环境，鼓励本地企业和民间资本投资兴业，推动非公经济快速健康发展。三是打造现代融资服务平台。积极引进民营银行、外资银行、地方国有银行和风险投资机构，探索建立乡镇投融资平台和发展区域性股份制村镇银行，支持发展小额贷款公司和担保公司。加强信用环境建设，支持商业银行创新金融产品，留住、激活、用好域内资金。促进城投公司做大做强，拓展经济建设投资、担保、咨询等业务，着力打造现代投融资集团。

　　（六）着力保障和改善民生，提高群众幸福指数。越是在经济困难时期，越要更加关注民生，保证民生财力不减、居民收入增幅不降。一是加快保障性住房建设。完成20114 套保障性住房建设，改造农村泥草房4000 户、危房400户。制定科学严密的分配方案，强化关键环节监管，严厉查处违规操作、弄虚作假、营私舞弊行为，做到住房分配公平合理，社区服务同步跟进。二是增强社会保障能力。提高企业离退休人员工资标准，提高失业保险支付标准，提高城镇居民医疗保险报销比例，扩大医疗保险门诊统筹范围。适度提高城乡低保补助标准，提高农村五保户供养标准，提高特困生救助标准。三是提高就业再就业水平。加强技能培训，强化就业服务，促进煤矿工人就业转型。大力发展商贸流通等服务业和中小微企业，扩大就业容量。鼓励支持高校毕业生、复员军人自主创业、自谋职业、基层就业。开发公益岗位150 个以上，安置就业困难人员。四是促进社会事业全面进步。着力改善办学条件，新建和改造4所学校，确保新一中10 月份投入使用。推进乡镇寄宿制学校建设，加快建设公办幼儿园。促进职业学院、技师学院、职业技术学校资源整合，积极打造本科教育、职业教育、技能培训为一体的综合性大学。改善医疗卫生服务条件，启动市妇幼保健院、残疾人康复中心建设，新建改造3 个社区卫生服务中心、2 个乡镇卫生院。新建扩建县区卫生监督所，推进食品药品检验检测中心建设。大力发展文化事业，利用现有文化设施，积极搭建文化产业发展平台。依托企业引进科研机构，大力培养实用型科技人才，增强企业自主创新能力。突出发展冰雪运动，广泛开展全民健身活动，积极举办各类体育赛事。扎实搞好精神文明建设，全面提高市民素质。加快广播电视、人口计生、妇女儿童、民族宗教等事业发展。五是切实解决民生热点难点问题。按照城市规划布局，调整优化公交运营线路，更新升级公交车辆，筹建北岸新城、金沙新区、新兴区、大同街东段停车场和调度中心，方便群众出行。落实弃管楼管理主体责任，细化接管方案，明确物业维修维护资金保障渠道，改善弃管楼环境。实施“暖房子”工程，完成40万平方米住宅楼外墙保温施工。推进市殡仪服务中心迁建，免收困难家庭四项基本殡葬服务费用。

　　（七）强力抓好安全稳定工作，推动和谐社会建设。突出解决影响安全稳定的主要矛盾，创造良好社会发展环境。一是全力抓好安全生产工作。加快推进地方煤矿资源整合、兼并重组，按照“大矿兼并小矿、小矿联合做大”资产重组的方式，本着同区域、同块段、同煤层资源整合的原则，形成一批集团化实体企业，淘汰一批不符合国家标准的煤矿。综合整治煤矿安全，控制住资金链和利益链，依法限制企业多头开户，开工矿井必须交足3000 万元以上的安全风险抵押金，严厉打击非法违法生产行为。严格落实“谁申报谁负责、谁审批谁负责、谁验收谁负责”制度，严把煤矿复产验收关。严格落实“四个责任”，实行驻矿监察组24 小时旁站式监管，实行煤矿投资者、矿长持证上岗制度，完善井下作业人员定位系统，保证矿井地质资料详实准确，做到严格兑规作业，全力促进安全生产形势好转。突出强化危险化学品生产、运输管理，严防发生爆炸、泄露等事故。全力抓好道路交通、消防、食品药品、易燃易爆、森林防火、特种设备等领域的安全工作。二是认真做好信访稳定工作。严格落实信访责任制，完善矛盾预警和排查调处机制，扎实开展领导接访下访活动，及时发现化解矛盾，力争将问题消灭在萌芽、解决在基层。严格信访事项办理、复查、复核三级程序，集中力量化解信访积案，依法处理违法信访行为。三是突出加强社会治安工作。完善立体化社会治安防控体系，推进传统治安管理向现代社会管理转变。强化网络舆情监管和情报信息研判预警，提高预防和应对各类突发事件能力。始终保持对违法犯罪的高压态势，有效遏制恶性案件发生。四是全面强化社会管理工作。健全完善“八大工作体系”，形成更加有效的社会服务管理网络，全面实行“农事村办、企事厂办、居民事社区办”制度，深入开展“联企、包区、助村、帮户”活动，突出解决“应该管但没人管”、“有人管但没管好”问题，重点解决群众“ 求助无门、投诉无果”问题。

　　三、全面加强政府自身建设

　　以提升公信力为目标，以优化环境为重点，勤勉作为，真抓实干，努力建设人民满意政府。

　　一是改进作风，抓好落实。全面落实中央“八项规定”，大力弘扬求真务实之风，严格兑现政府承诺，坚决做到事事有着落，件件有结果。着力提高决策执行能力，强化重大决策、重点项目、重要工作的督办落实，确保一项工作抓到底，一件事情抓出头。注重提高执行效率，坚决做到政令畅通、令行禁止。改进文风会风，少开会、开短会，讲短话、讲管用的话，腾出更多精力抓落实。大力倡导艰苦奋斗、厉行节约之风，严格控制行政经费支出，力戒奢侈浪费。

　　二是优化环境，保障发展。集中清理行政审批项目，失去法律依据的一律取消，优化保留事项的审批流程，能转为备案的一律改为备案，做到审批程序最优、效率最高。集中清理行政事业收费项目，能取消的坚决不收，必须收取的明确收费额度，做到收费项目最少、标准最低。从严从重从快打击侵害投资者权益行为，依法解决投资者的各类经济纠纷，加强社会信用体系建设，推行失信企业黑名单制，严厉打击恶意逃废债务行为，打造公平正义的司法环境。制定实施扶持企业健康发展政策，帮助企业渡难关、保生存、稳发展。

　　三是依法行政，规范用权。严格按照法定权限和程序履职尽责，切实规范行政执法行为。认真执行市人大及其常委会的决议、决定，主动接受人大依法监督和政协民主监督，及时办理人大代表议案、意见、建议和政协委员提案。坚持问政于民、问计于民、问需于民，提高决策科学化、民主化、法治化水平。大力推进政务公开，健全信息发布制度，提高工作透明度。

　　四是廉洁从政，取信于民。严格落实党风廉政建设责任制，全面加强惩防腐败体系建设。突出强化保障性住房分配、安全生产等重点领域监管，严肃查处弄虚作假、失职渎职、充当保护伞等违纪违法行为，着力解决人民群众反映强烈的问题，以廉洁、阳光、诚信的良好形象，提升政府公信力。

　　各位代表，新的任务、新的挑战、新的使命，要求我们更加团结一心、奋发有为。让我们在省委、省政府和市委的领导下，坚定信心，迎难而上，共同开创“加快经济崛起，建设幸福之城”的新局面！
